# Cyber Hygiene Methodology for Raising Cybersecurity and Data Privacy Awareness in Health Care Organizations: Concept Study

**DOI:** 10.2196/41294

**Published:** 2023-07-27

**Authors:** Elina Argyridou, Sokratis Nifakos, Christos Laoudias, Sakshyam Panda, Emmanouil Panaousis, Krishna Chandramouli, Diana Navarro-Llobet, Juan Mora Zamorano, Panagiotis Papachristou, Stefano Bonacina

**Affiliations:** 1 KIOS Research and Innovation Center of Excellence, University of Cyprus Nicosia Cyprus; 2 Health Informatics Centre Department of Learning, Informatics, Management and Ethics Karolinska Institutet Stockholm Sweden; 3 Internet of Things and Security Centre University of Greenwich London United Kingdom; 4 Department of Research and Innovation, Fundacio Privada Hospital Asil de Granollers Barcelona Spain; 5 Instituto de Invest, Sanitaria Puerta de Hierro, Servicio Madrileno de Salud , Majadahonda Madrid Spain; 6 Division of Family Medicine and Primary Care, Department of Neurobiology, Care Sciences and Society Karolinska Institutet Stockholm Sweden

**Keywords:** cyber hygiene, cybersecurity, awareness, training, health care, risk management, mobile phone

## Abstract

**Background:**

Cyber threats are increasing across all business sectors, with health care being a prominent domain. In response to the ever-increasing threats, health care organizations (HOs) are enhancing the technical measures with the use of cybersecurity controls and other advanced solutions for further protection. Despite the need for technical controls, humans are evidently the weakest link in the cybersecurity posture of HOs. This suggests that addressing the human aspects of cybersecurity is a key step toward managing cyber-physical risks. In practice, HOs are required to apply general cybersecurity and data privacy guidelines that focus on human factors. However, there is limited literature on the methodologies and procedures that can assist in successfully mapping these guidelines to specific controls (interventions), including awareness activities and training programs, with a measurable impact on personnel. To this end, tools and structured methodologies for assisting higher management in selecting the minimum number of required controls that will be most effective on the health care workforce are highly desirable.

**Objective:**

This study aimed to introduce a cyber hygiene (CH) methodology that uses a unique survey-based risk assessment approach for raising the cybersecurity and data privacy awareness of different employee groups in HOs. The main objective was to identify the most effective strategy for managing cybersecurity and data privacy risks and recommend targeted human-centric controls that are tailored to organization-specific needs.

**Methods:**

The CH methodology relied on a cross-sectional, exploratory survey study followed by a proposed risk-based survey data analysis approach. First, survey data were collected from 4 different employee groups across 3 European HOs, covering 7 categories of cybersecurity and data privacy risks. Next, survey data were transcribed and fitted into a proposed risk-based approach matrix that translated risk levels to strategies for managing the risks.

**Results:**

A list of human-centric controls and implementation levels was created. These controls were associated with risk categories, mapped to risk strategies for managing the risks related to all employee groups. Our mapping empowered the computation and subsequent recommendation of subsets of human-centric controls to implement the identified strategy for managing the overall risk of the HOs. An indicative example demonstrated the application of the CH methodology in a simple scenario. Finally, by applying the CH methodology in the health care sector, we obtained results in the form of risk markings; identified strategies to manage the risks; and recommended controls for each of the 3 HOs, each employee group, and each risk category.

**Conclusions:**

The proposed CH methodology improves the CH perception and behavior of personnel in the health care sector and provides risk strategies together with a list of recommended human-centric controls for managing a wide range of cybersecurity and data privacy risks related to health care employees.

## Introduction

### Background

According to the technical series published by the World Health Organization on primary health care [1], information and communications technologies are nowadays very common with the introduction of smartphones, tablets, and laptop computers. On the one hand, such technologies have resulted in a positive impact on patient care with the increasing growth of electronic health records. However, such medical databases, which often include personal information and financial data, among others, have become a target for cyberattacks.

The origins of cybercrime can be traced back to the late 1970s as the computer IT industry took shape. What began as spam eventually transitioned to computer viruses and malware (eg, WannaCry). Inevitably, the rise of cybersecurity incidents is a growing threat to the health care industry in general and to hospitals in particular [2]. Although the impact of cybersecurity is not unique to the health care industry, concerted efforts to protect health care stakeholder data have lagged in comparison with those of other industries [3]. With the fast digitization of patient health records, the impact of data breaches on hospitals has caused major economic and intangible damage. To counteract the impact of cyberattacks, organizations have adopted governance strategies to promote best practices for securing the electronic infrastructure of hospitals and other clinical environments [2,4].

Existing cybersecurity practices in health care organizations are insufficient [2-4] and have affected the integrity of medical data and the confidentiality of patients. Even with increasing instances and case studies of cyberattacks within health care organizations, many institutions still remain ignorant of cybersecurity and rely on legacy systems such as Windows XP and Windows NT 3.1 despite the warning from relevant vendors such as Microsoft who have stated that security updates and support have been withdrawn for such systems, presenting a security risk. One of the main reasons for health care organizations becoming an attractive target for cybersecurity attacks is the large volume of personal data being handled, which present an economic value in the black market [5]. The weaker security posture in health care organizations is mainly because of the lack of a cybersecurity budget, which results in minimal access to technology and expertise. Morgan [6] observed that the health care industry will respond by spending US $125 billion cumulatively from 2020 to 2025 to strengthen its cyber defenses. Such an investment in cyber defense is necessitated by the number of attacks, which have increased 5-fold since the COVID-19 pandemic [7]. Such incidents have been recently witnessed within the Health Service Executive of Ireland [8]. A similar case was reported in August 2021, when a ransomware attack was launched against the COVID-19 vaccination booking system in the region of Lazio, Italy [9].

Traditionally, health care organizations have not considered an investment in cybersecurity as necessary as the focus has predominantly been on providing patient care and people believed that there would be no motivation to attack these organizations. However, recent findings have shown that health care data are considerably more valuable than any other data. In contrast, the increasing use of Internet of Things technologies in health care has increased the attack surface from information security to physical safety. Following the increasing familiarity and convenience of using single digital devices for both personal and professional activities, Wani et al [10] noted that major challenges to the health care IT infrastructure stem from the use of devices with insufficient security controls by hospital staff and the lack of control or visibility for management to maintain security requirements. Additional factors such as the lack of awareness among hospital staff, lack of direction or guidance for secure use of Bring Your Own Device or Phone, poor user experience, lack of compliance with legal requirements for accessing secure health care IT systems, shortage of cybersecurity skills, and loss of devices are also cited as causes for security threats. Despite advances in the field of IT systems to enhance the overall security of health care organizations, critical challenges remain owing to the lack of emphasis on human factors in cybersecurity.

As a substantial proportion of cyberattacks are directed toward users through deceptive means such as spam emails and application masquerading, users play a critical role in cybersecurity alongside technical controls. This is particularly the case in health care as deceiving a nurse, physician, health care IT professional, or administrator can affect the privacy and physical safety of patients. For example, Saxon et al [11] demonstrated that implantable medical devices (eg, pacemakers and cardioverter-defibrillators) are susceptible to adversarial interference (remotely) that not only violates the integrity and confidentiality of patients’ data and medical telemetry but could also compromise patients’ physical safety.

In a recently published systematic survey by Nifakos et al [12], the authors concluded that there is a fundamental paradigm shift from targeting IT infrastructure as a vulnerability of a health care organization to focusing on human vulnerability, which relies on the existing IT infrastructure. One of the key observations of the authors relates to the crossover of personal information between social media use by health care professionals, which has proved to be a successful source of information for launching targeted phishing attacks. Jalali and Kaiser [13] conducted research on the factors affecting employee decision-making that enable them to click on phishing links. This study focused on clicking behavior, which was analyzed using the Theory of Planned Behavior. The authors concluded that there is a strong correlation between employee workload and the behavior of noncompliance while responding to phishing attacks. As a result of the systematic survey, Nifakos et al [12] proposed implementing training modules to promote the use of privacy-setting options provided by social media platforms. The authors also acknowledged that there needs to be a targeted organizational program to undertake cyber risk and privacy impact assessment leading to the identification of potential health care infrastructure vulnerabilities. Such a program should place a high degree of emphasis to consider a human-centric approach. In addition, the authors acknowledged the fact that, despite several organizations and researchers having identified the need for delivering cybersecurity training to health care professionals, there is little consensus on the curriculum and systematic methodology for evaluating the impact of cyber hygiene (CH). This review presented in detail several case studies that are often experienced by health care professionals, including frontline medical staff, nurses, management teams, and hospital administrators.

In the past, studies on security training in health care have investigated offering education to health care professionals aimed at gaining awareness of digital applications and platforms for increasing knowledge on health care data privacy and security risks [14]. The authors further highlighted the factors to consider while designing training and awareness programs for health care personnel. The extent to which security training and awareness programs work for different users has been studied from multiple angles. Heartfield et al [15] have shown that, against deception-based attacks such as semantic social engineering, self-study and work-based training are considerably more effective than formal education in cybersecurity. Meanwhile, Wash and Cooper [16] have indicated that training materials from security experts, third-party organizations, and peers can also positively influence cybersecurity practices.

Among the several barriers and reluctance to adopt several recommendations that have been summarized in the literature identified within the health care sector [12], the lack of funding dedicated to securing the IT infrastructure of health care organizations has been cited as a critical limitation. In contrast to other digital industrial sectors such as finance, banking, and media, the main driver of revenue is the successful delivery of health care services. In addition, as all health care services are delivered to patients by humans, the financial structure of health care organizations aims to prioritize expenses to retain human capital. This phenomenon is reflected in the lack of a *Chief Information Security Officer*, who serves as an official member of several health care boards.

Despite the economic challenges often encountered by health care organizations, there is recently increasing evidence of investment, determined by hospital management to strengthen IT infrastructure [4]. Although some of these endeavors might be voluntary, the data governance policies enacted by national authorities have also acted as a catalyst for increasing cybersecurity budgets. However, the successful adoption of digital transformation strategies within the health care industry relies on the successful acceptance among health care professionals of addressing the risks posed by cyber threats. Thus, it is important to deliver *awareness* and *training* programs to health care professionals. The role of human behavior in coping with cyberattacks and strengthening cyber defenses is grouped into the theme of “human factor” in cybersecurity.

In general, health care organizations strive to apply cybersecurity and data privacy guidelines that focus on the human factor. This is because general guidelines are typically hard to map to specific controls (eg, awareness activities and training programs) with a proven positive effect on personnel while avoiding overspending by implementing unnecessary or less relevant controls. Currently, there is a lack of tools and methodologies for assisting higher management in selecting the necessary controls that will have the greatest impact on health care personnel. This is the key challenge that the proposed CH methodology addresses.

### Objectives

**Figure 1:**
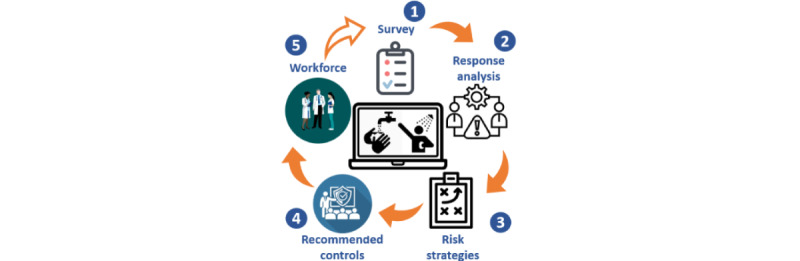
Outline of the exploratory cyber hygiene methodology based on survey data and risk assessment.

The exploratory CH methodology is outlined in Figure 1 and comprises 5 steps (Textbox 1).

**The 5 steps of the exploratory cyber hygiene methodology.**

**Knowledge extraction through a survey questionnaire**

- This step involves extracting knowledge and assessing the needs and gaps of different employee groups at health care organizations through a set of questions in a survey questionnaire.

**Response processing and analysis**

- In this step, the responses collected from the participants are processed and analyzed to evaluate the cybersecurity and data privacy risks and quantify them through their risk marking.

**Risk strategy identification**

- In this step, the most effective strategy to manage each risk is identified.

**Recommendation of controls**

- In this step, human-centric controls that are mapped in advance with a specific strategy are recommended to implement the identified strategy.

**Application of the controls to the personnel**

- In this final step, the management team can apply the controls to the workforce to improve the level of cybersecurity and data privacy awareness.

The empty arrow that closes the loop from step 5 to step 1 in Figure 1 indicates future work: running the survey again after some time to confirm that the situation in terms of cybersecurity and data privacy awareness has improved after the application of the recommended controls.

## Methods

### Study Design

A cross-sectional, exploratory survey study together with a proposed risk-based survey analysis approach were designed and deployed to describe CH awareness within 3 health care organizations participating in the Secure and Private Health Data Exchange (CUREX) project.

### Survey Construction

**Figure 2:**
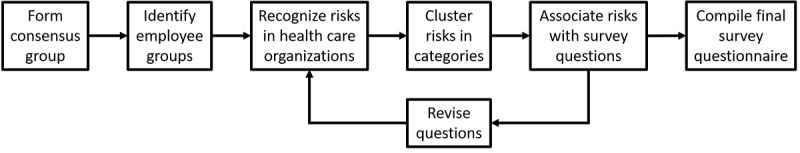
Diagram of the procedure to construct the survey.

The survey was designed to capture different aspects of employee awareness of cybersecurity, data privacy and data protection, employee training, and use of connected devices. The procedure to construct the survey is illustrated in the diagram in Figure 2. First, the working group for the CUREX project together with representatives from all 3 health care organizations formed a consensus group that performed an initial review of existing literature and resources on CH, including relevant documents by health care agencies such as the US Department of Health and Human Services that highlight the top threats in this sector [17]; reports and recommendations by international cybersecurity organizations and centers such as the European Union Agency for Cybersecurity [18-21], the European Cyber Security Organisation [22], and the Center for Internet Security (CIS) [23]; and previous surveys on this topic [24,25]. The consensus group consisted of 16 members with a differential background ranging from IT and cybersecurity expertise and technical and medical academia to health care professionals. Next, the consensus group identified the main employee groups in health care organizations (ie, administrative, medical and clinical, IT and technical, and executive and security personnel; see the *Study Population* section for details). The intuition is that employee groups are not equally vulnerable to cybersecurity threats as they have varying awareness levels of cybersecurity and data privacy and undertake daily tasks that do not expose them to the same risks. Next, after consulting the representatives from the 3 health care organizations, various risks were recognized (eg, employees not being aware of cyber threats in the health care sector, not considering cybersecurity during daily work, and not knowing about internal security procedures), followed by clustering in representative risk categories; see the risk categories and descriptions in Table 1. An initial list of questions was then prepared and associated with each risk category aiming to quantify the relevant risk according to the responses. The questions were reviewed and refined in multiple iterations. The process was repeated when new risks were recognized, leading to new risk categories or the adaptation of previously defined categories, followed by the addition of other questions. Finally, after several review rounds, the consensus group concluded on a final survey with a total of 28 questions; see the list of questions in Multimedia Appendix 1. The survey questions were prepared in English, later translated into the native languages of the health care organizations in the CUREX project, and administered in all 3 languages.

**Table 1:** Risk categories for all employee groups.

Risk category	Survey questions	Risk description
Cyber hygiene	2, 3, and 4	Not aware of what cyber hygiene is
Cybersecurity awareness	8, 11, and 13	Not aware of cybersecurity threats in health care and related incidents
Data privacy and protection awareness	5, 6, 8, 12, and 14	Not aware of what GDPR^a^ is or of data privacy and protection threats in health care and related incidents
Cybersecurity training	9, 15, 17, and 20	Not attending existing training, not considering cybersecurity during daily work, not knowing about internal procedures for cybersecurity threats, and limited knowledge about cybersecurity (self-assessed)
Data privacy and protection training	7, 10, 16, 18, 19, and 21	Not attending existing training, not considering data privacy during daily work, not knowing about internal procedures for data privacy threats and who is responsible for data protection, managing personal data frequently, and limited knowledge about data privacy (self-assessed)
Communication channels	22, 23, and 24	Limited number of communication channels that are available in the organization or preferred by employees and limited communication with IT personnel
Secure connection and use of devices	25, 26, 27, and 28	Not aware of or not following policies, guidelines, or best practices regarding remote connection, using public access networks, using personal devices (BYOD^b^), and using personal USB sticks

^a^GDPR: General Data Protection Regulation.

^b^BYOD: Bring Your Own Device.

Mapping the CH landscape by reviewing existing literature and studying the available resources was fundamental for compiling the final survey questionnaire. For instance, driven by the list of top threats in health care, specific questions were included to reveal how familiar the employees working in this sector are with these threats, whether they are aware of relevant incidents both inside and outside their organization, whether they are able to recognize such incidents at the early stages, and how confident they feel to handle them. In addition, given the nature of these top threats, it was decided to define the risk categories and the associated survey questions in such a way that there was a clear distinction between cybersecurity and data privacy risks.

Finally, the recommendations by cybersecurity agencies and organizations were reflected in several risk categories and associated survey questions. These recommendations included (1) raising cybersecurity awareness; (2) securing medical and portable devices, including Bring Your Own Device and Bring Your Own App schemes; (3) securing physical access and health information; and (4) educating users against social engineering attacks (eg, phishing emails).

### Study Population

In each health care organization, we identified four main employee groups that were eligible for the survey study: (1) administrative (eg, administration manager, secretary, reception, call center, and human resources), (2) medical and clinical (eg, department and unit manager, physician, and nurse), (3) IT and technical (eg, IT manager, IT staff, and software developer), and (4) executive and security (eg, director, subdirector, hospital manager, chief information security officer, chief security officer, and data protection officer).

The first 2 groups (ie, the *administrative* and *medical and clinical* groups) typically had more employees compared with the *IT and technical* and *executive and security* groups.

The participation of users in the study was carried out through a systematic recruitment process, which was launched using either proprietary (such as internal e-learning tools or through email campaigns) or open web-based survey tools (eg, the EUSurvey tool). Additional channels of recruitment included participation through emails and the use of existing e-learning platforms, which were often used by hospital physicians for medical learning and other events. The user recruitment process was conducted between mid-June 2020 and the end of September 2020. All participants had access to the survey preamble with information on the purpose of the survey. All participants provided digital consent before proceeding with the survey questions. Duplicate entries were avoided by preventing users with the same IP address from accessing the survey twice.

The CI based on the survey responses with respect to the population size was determined by *P*<.05 (95% CI). For the calculation, a web-based CI calculator was used [26]. More specifically, the following formula was used:



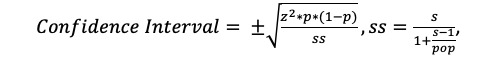



where *z* is the value of the confidence level (eg, 1.96 for 95% CI), *p* is the percentage of picking a choice expressed as a decimal (eg, 0.5), *s* is the sample size (ie, number of responses), and *pop* is the population size.

### Risk Categories

The survey questions were grouped into 7 risk categories based on their topic and structure, as shown in Table 1, which facilitated risk analysis and profiling of each employee group. The first column provides the name of the risk category, the second column lists the number of the associated survey questions, and the third column describes the risks of the corresponding category.

### Survey Questions

Each of the 28 survey questions could be a single- or multiple-answer question. In addition, the survey questions had one of the following Likert-type responses to describe the extent of the respondents’ awareness, agreement, frequency of use (adoption of CH practices), knowledge, and satisfaction: (1) *yes, no, or I don’t know* (awareness), (2) 1=*I strongly disagree* to 5=*I strongly agree* (agreement), (3) 1=*Never* to 5=*In every daily activity* (frequency of use), (4) 1=*I have no knowledge* to 5=*I am an expert* (knowledge), and (5) 1=*Very disappointing* to 5=*Very satisfying* (satisfaction).

Among the different types of questions outlined previously, the scale of 1 represents the lowest value, whereas 5 represents the highest value of the impact that corresponds to each question. On the basis of these marks, we described the awareness and understanding of CH for each respondent as well as each employee group in total. Each of the employee group members was asked to go through identified statements to determine their awareness and relevance in terms of the survey purpose on a scale of 1 to 5, where 1 meant no awareness or relevance and 5 meant high awareness or relevance. On the basis of the responses collected from the participants, we described the extent of awareness and relevance for each employee group and suggested an appropriate strategy and associated controls for raising cybersecurity and data privacy awareness.

### Risk-Based Survey Analysis Approach

#### Overview

In this section, we describe a proposed risk-based approach for the analysis of the survey responses. The aim was to design effective processes to increase awareness and training on CH. These processes include *identification*, *analysis*, *monitoring*, *evaluation,* and *treatment* of various risks that each health care organization may have. By applying these risk processes, we described the risks using a risk matrix with a scoring system (1-5). Then, we proceeded with evaluating the risks, which pointed to specific risk strategies for managing the associated risks toward raising the cybersecurity and data privacy awareness of different employee groups. Risk strategies were mapped to a recommended subset of controls to manage the risks of each employee group.

#### Risk Strategies

The *impact-probability risk matrix* is shown in Table 2. This matrix has 2 dimensions, namely, the *risk probability*, which shows the likelihood of a risk to happen, and the *risk impact,* which shows the importance and severity of the risk.

By multiplying the risk probability by the risk impact, we obtained the risk evaluation marking that shows whether the risk is low, medium, or high.

The *risk evaluation matrix*, which points to specific risk strategies based on the risk evaluation marking, is shown in Table 3. Each risk strategy corresponds to specific controls for *mitigating*, *reducing*, *monitoring*, *checking*, and *accepting* risks. For instance, when the risk is higher, the trainings should be more often (eg, weekly) starting from basic information (eg, beginner-level material). In a similar fashion, when the risk is lower, the trainings could be less frequent (eg, monthly or quarterly), including more details (eg, advanced-level material). Finally, if the risk is very low, it is acceptable, and the employees are acknowledged and rewarded for following good CH practices.

**Table 2:** Impact-probability risk matrix.

Risk probability	Risk impact				
	Negligible (1)	Minor (2)	Moderate (3)	Considerable (4)	Severe (5)
Very likely (5)	Low-medium	Medium	Medium-high	High	High
Likely (4)	Low	Low-medium	Medium	Medium-high	High
Possible (3)	Low	Low-medium	Low-medium	Medium	Medium-high
Unlikely (2)	Low	Low	Low-medium	Low-medium	Medium
Very unlikely (1)	Low	Low	Low	Low	Low-medium

**Table 3:** Risk evaluation matrix.

Risk marking	Risk evaluation	Risk strategy	High-level action plan
20-25	High	Mitigation	Mitigate the risk: improve skills, raise awareness, monthly or weekly actions for beginner level
15-19	Medium-high	Reduction	Reduce the risk: improve skills, raise awareness, quarterly or monthly actions for intermediate level
10-14	Medium	Monitoring	Monitor the risk: increase awareness, semiannual or quarterly actions for intermediate or advanced level
5-9	Low-medium	Checking	Check the risk: retain awareness, annual or semiannual interventions for advanced level
1-4	Low	Acceptance	Accept the risk: acknowledgment and rewards

#### Risk Procedures

To use the risk evaluation matrix (Table 3), in this section, we define the *risk impact* and *risk probability*.

In Table 4, the risk impact is defined using a scoring system from 1 to 5 based on the structure of the survey questions. The lowest risk impact has the lowest mark (1), whereas the highest impact has the highest mark (5). Medium marks (2-4) indicate medium risk impacts.

In Table 5, the risk probability is defined based on the total number of responses. When the total number of responses is high, the likelihood of the risk to happen is higher, whereas when the total number of responses is low, the likelihood is low.

To calculate the *risk marking*, we applied the following formula:



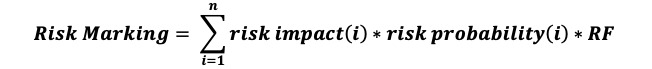



where *i*=1,..., *n* is the number of responses and RF is the risk factor.

For the risk factor, the following formula is applied: *Risk Factor = 5 / (NoQ) × (NoR)*, where *NoQ* is the total number of questions in each risk category and *NoR* is the total number of responses of each employee group from each organization. The number 5 is chosen as the maximum mark of the scoring system so that we can reach the highest level of possible risk.

An example risk marking calculation as part of the risk-based approach is provided in the *Results* section.

**Table 4:** Risk impact definition for different types of survey questions.

Risk impact number	Risk impact	Frequency	Agreement	Knowledge	“Yes,” ”no,” or “I don’t know”	Multiple answers
1	Low	Daily	Strongly agree	In depth	“Yes”	All selected
2	Low-medium	Weekly	Agree	Very well	N/A^a^	Many selections
3	Medium	Monthly	Cannot say	Well	“I don’t know”	Enough selections
4	Medium-high	Rarely	Disagree	Heard of it	N/A	Few selections
5	High	Never	Strongly disagree	Never heard of it	“No”	One or nothing

^a^N/A: not applicable.

**Table 5:** Risk probability definition.

Risk probability number	Risk probability	Re^a^
1	Very unlikely	0-Re × (1/5)
2	Unlikely	Re × (1/5)-Re × (2/5)
3	Possible	Re × (2/5)-Re × (3/5)
4	Likely	Re × (3/5)-Re × (4/5)
5	Very likely	Re × (4/5)-Re

^a^Re: number of responses.

### Ethical Considerations

According to the Swedish Ethical Review Authority, studies that do not collect any sensitive information from the human participants (nonpatients) according to section 3 do not fall under the obligations of the Swedish Ethical Review Act (EPL 2003:460). All participating human subjects (health care professionals) were anonymous survey respondents and provided their informed consent prior to survey response. No sensitive personal data was collected. An ethical approval was not considered.

### Rationale of Human-Centric Controls

To implement the high-level action plans for the risk strategies shown in Table 3, we need relevant human-centric controls (ie, measures and interventions; the single term “controls” will be used hereunder to include measures, controls, and interventions) to be associated with each risk strategy. As most resources recognize training and awareness campaigns as key prerequisites in increasing awareness and achieving a common understanding of cyber threats and security risks at all hierarchical levels, the proposed list includes targeted *Training* and *Awareness* controls. As the use of rewards has been reported in the literature to be beneficial in encouraging and motivating employees to adopt desirable behaviors [27,28], our list also includes *Motivation* and *Reward* controls.

In particular, a subset of the proposed controls is inspired by the subcontrols presented in the CIS report “CIS Control 17: Implement a Security Awareness and Training Program v7.1” [29]. These controls are properly adapted to the objectives of CH within the European Union–funded Horizon 2020 project CUREX [30] (eg, there are separate controls for cybersecurity and data privacy). Notably, the CIS controls report version 8 released in May 2021 includes the control “Conduct Role-Specific Security Awareness and Skills Training,” which is captured in our approach through the consideration of different employee groups. Some motivation controls are adopted to incorporate the notion of nudges that are proposed in the Secure Behavior Nudging Tool [31] developed in the context of the Horizon 2020 Protection and Privacy of Hospital and Health Infrastructures with Smart Cyber Security and Cyber Threat Toolkit for Data and People project [32]. In general, nudges are behavioral interventions that usually take place in a timely manner (ie, during daily work) rather than in “out-of-context” training in the classroom. Finally, additional controls were introduced by the CUREX research team and inspired by guidelines and good practices applied across various domains. These include awareness controls (eg, inclusion of cybersecurity and data privacy in the agenda of each meeting that takes place in the health care organization) and reward controls that are intended mainly to acknowledge employees who behave responsibly and celebrate desirable practices within the organization on various occasions.

### Selecting Controls for Each Risk Strategy

The main idea for selecting controls that are relevant to each risk strategy (Table 3) is as follows. As a risk increases and the risk strategy changes from *Acceptance* to *Checking* to *Monitoring* to *Reduction* and, finally, to *Mitigation*, the applied controls should move from *Rewarding* to *Motivation* to *Awareness* and, finally, to *Training* controls to address and properly manage the risk. The intuition is that, for example, the *Motivation* controls assume some level of awareness to be effective; thus, these controls cannot help in the case of *Reduction* or *Mitigation* risk strategies, where a lack of awareness or knowledge is observed. Moreover, the *Training* controls are expected to have a larger impact on managing the risk when they are combined or applied after *Awareness* controls. In addition, moving from *Monitoring* to *Reduction* and, finally, to *Mitigation* implies that the frequency of the *Awareness* and *Training* controls should be increased so that the employees are more frequently exposed to the awareness messages and training material. In contrast, the content level of awareness and training should be decreased (eg, beginner-level content is more appropriate in the *Mitigation* risk strategy, whereas advanced-level content better fits the *Monitoring* risk strategy as the employees have a baseline awareness or knowledge of the corresponding risk).

## Results

### Candidate Human-Centric Controls

For this study, we created a list of 19 candidate controls, C1 to C19, which are listed in Table 6, followed by the association of controls with each risk category, shown in Textbox 2.

The candidate controls in Table 6 are categorized as follows: (1) training controls (C1, C2, C6, C7, C8, C9, C10, and C11), (2) awareness controls (C3, C4, C5, C12, and C13), (3) motivation controls (C14, C15, and C17), and (4) rewarding controls (C16, C18, and C19).

Obviously, not all controls are appropriate for all risk categories shown in Table 3 for all employee groups. However, note that a specific control may be relevant to multiple risk categories. To this end, each risk category has a list of associated controls, and either all or a subset of the associated controls can be applied as part of the identified risk strategy, as shown in Textbox 2.

Moreover, different *implementation levels* can be considered for an individual control. For instance, a control that is related to training (eg, C2 and C6) or a control that implements an awareness program and updates its content (eg, C3, C4, and C5) may have varying *implementation levels*, for example, *frequency* (ie, weekly, monthly, quarterly, semiannually, or annually), *content level* (ie, beginner, intermediate, or advanced level), and *target audience* (ie, administrative, medical and clinical, IT and technical, and executive and security personnel). These implementation levels can be properly selected for the identified risk strategy depending on the employee group.

**Table 6:** Candidate human-centric controls.

Number	Control title	Control description	Related resource
C1	Perform a skill gap analysis	Perform a skill gap analysis to understand the skills and behaviors that employees are not adhering to; using this information to build a baseline education road map	CIS^a^ subcontrol 17.1
C2	Deliver training to fill the skill gap	Deliver training to address the skill gap identified to positively affect employees’ security behavior	CIS subcontrol 17.2
C3	Implement a cybersecurity awareness program	Create a cybersecurity awareness program for employees to ensure that they understand and exhibit the necessary behaviors and skills to help ensure the security of the organization	CIS subcontrol 17.3
C4	Implement a data privacy awareness program	Create a data privacy awareness program for employees to ensure that they understand and exhibit the necessary behaviors and skills to help ensure the security of the organization	CIS subcontrol 17.3
C5	Update awareness content frequently	Ensure that the organization’s security awareness program is updated frequently to address new technologies, threats, standards, and business requirements	CIS subcontrol 17.4
C6	Train workforce on secure authentication	Train employees on the importance of enabling and using secure authentication	CIS subcontrol 17.5
C7	Train workforce on identifying social engineering attacks	Train employees on how to identify different forms of social engineering attacks such as phishing, phone scams, and impersonation calls	CIS subcontrol 17.6
C8	Conduct mock social engineering exercises	Conduct mock social engineering attacks (phishing, phone scams, and impersonation calls) to assess the readiness and response level of the employees	CIS subcontrol 17.6
C9	Train workforce on sensitive data handling	Train employees on how to identify and properly store, transfer, archive, and destroy sensitive information	CIS subcontrol 17.7
C10	Train workforce on causes of unintentional data exposure	Train employees to be aware of causes of unintentional data exposure, such as losing their mobile devices or a USB stick with sensitive data and emailing the wrong person	CIS subcontrol 17.8
C11	Train workforce members on identifying and reporting incidents	Train employees to be able to identify the most common indicators of an incident and report such an incident	CIS subcontrol 17.9
C12	Include cybersecurity in the meeting agendas	Set cybersecurity as a standing agenda item at meetings	CUREX^b^ project
C13	Include data privacy in the meeting agendas	Set data privacy as a standing agenda item at meetings	CUREX project
C14	Introduce nudges to motivate cybersecurity behaviors	Introduce nudges as behavioral interventions to motivate and encourage employees to adopt desirable cybersecurity behaviors that they are already aware of	PANACEA^c^ project
C15	Introduce nudges to motivate data privacy behaviors	Introduce nudges as behavioral interventions to motivate and encourage employees to adopt desirable data privacy behaviors that they are already aware of	PANACEA project
C16	Acknowledge employees who behave in a cybersecurity- and data privacy–responsible way	Acknowledge employees who demonstrate cybersecurity and data privacy behaviors (eg, report scam emails and suspicious incidents to the IT department) and reward them (eg, introduce awards such as “Cybersecurity Employee of the Year”)	CUREX project
C17	Introduce a cybersecurity and data privacy champion role	Nominate an employee within each department or team in the organization who, given some specific skills and knowledge, will be responsible for promoting cybersecurity and data privacy best practices in daily work	CUREX project
C18	Celebrate cybersecurity awareness on specific occasions	Introduce a specific day, week, or month during the year for celebrating cybersecurity (eg, the NCSAM^d^ observed in the United States and the ECSM^e^, both celebrated in October)	CUREX project
C19	Celebrate data privacy and protection awareness on specific occasions	Introduce a specific day, week, or month during the year for celebrating data privacy and protection (eg, the Data Privacy Day in the United States and the European Data Protection Day, both observed every January 28)	CUREX project

^a^CIS: Center for Internet Security.

^b^CUREX: Secure and Private Health Data Exchange.

^c^PANACEA: Protection and Privacy of Hospital and Health Infrastructures with Smart Cyber Security and Cyber Threat Toolkit for Data and People.

^d^NCSAM: National Cybersecurity Awareness Month.

^e^ECSM: European Cybersecurity Month.

**Recommended controls for the risk categories of all employee groups.**

**Cyber hygiene**

- C3, C4, C5, C12, C13, C16, C17, C18, and C19

**Cybersecurity awareness**

- C3, C5, C11, C12, C16, C17, and C18

**Data privacy and protection awareness**

- C4, C5, C11, C13, C16, C17, and C19

**Cybersecurity training**

- C1, C2, C7, C8, C11, C12, C14, C16, C17, and C18

**Data privacy and protection training**

- C1, C2, C9, C10, C11, C13, C15, C16, C17, and C19

**Communication channels**

- C3, C4, C5, C14, C15, and C17

**Secure connection and use of devices**

- C3, C4, C5, C6, C9, C10, C14, C15, C16, C17, C18, and C19

### Mapping of Controls to Risk Strategies

An indicative mapping of candidate controls (Table 6) with respect to risk strategies (Table 3) and risk categories (Table 1) is shown in Table 7.

For instance, for the *Cyber hygiene* risk category, as we move from the *Acceptance* risk strategy to the *Mitigation* risk strategy, more aggressive and effective controls are recommended to be applied for addressing the increasing risk. In this case, controls C3 and C4, which are related to the implementation of a cybersecurity and a data privacy awareness program, respectively, can be implemented monthly or weekly (ie, frequency level) for beginners (ie, content level) in the *Mitigation* risk strategy as the personnel is completely unfamiliar with CH, whereas in the *Reduction* risk strategy, the awareness programs can be implemented quarterly or monthly with intermediate-level content as the employee group has some basic knowledge of what CH is.

In the case of the “Cybersecurity Training” risk category, as shown in Table 7, controls C1 and C2, related to the analysis and filling of the skill gap, could be used only in the *Mitigation* risk strategy as the employee group probably lacks basic cybersecurity skills (eg, selecting a strong password) and has limited knowledge about cybersecurity. In contrast, controls C7 and C8, related to training for identifying social engineering attacks (eg, in person, over the phone, or through phishing emails) and conducting mock social engineering exercises (eg, fake phishing emails sent out by the organization’s IT department), are recommended for both the *Mitigation* and *Reduction* risk strategies. This is because social engineering is probably the most serious threat that the health care workforce needs to defend against. Again, the frequency (ie, monthly or weekly vs quarterly or monthly), content level (eg, baseline phishing emails for beginners vs more sophisticated phishing emails with email address spoofing), and target audience are adapted according to the risk strategy.

For these 2 risk categories, listed in Table 7, the *Monitoring* risk strategy may include mild controls (eg, C12 and C17) for discussing cybersecurity in internal meetings and assigning a cybersecurity champion in the team and department to monitor the situation, or in the case of the *Cybersecurity Training* risk category, the strategy may include training on identifying and reporting incidents (ie, C11) other than social engineering attempts. This is because, for this risk strategy, the employee group can be assumed to have adequate knowledge of social engineering attacks and how to recognize and defend against them and inform their IT department; thus, the focus should be on different types of suspicious events or behaviors. In addition, for the *Cybersecurity Training* risk category, the *Monitoring* risk strategy may also include nudges (eg, to encourage updating more often and choosing stronger passwords for their accounts) as this risk strategy assumes some level of awareness and basic knowledge of the underlying threats, and nudges aim to motivate desirable cybersecurity behaviors to further reduce the associated risk. Finally, for the *Checking* and *Acceptance* risk strategies, where the corresponding risk can be considered tolerable, the recommended controls include mainly acknowledging and rewarding desirable and “good example” behaviors by individuals or teams within the health care organization as well as celebrating cybersecurity event occasions.

**Table 7:** Indicative mapping of controls to risk strategies for the risk categories of all employee groups.

Risk category	Risk strategy				
	Mitigation	Reduction	Monitoring	Checking	Acceptance
Cyber hygiene	C3, C4, C5, C12, and C13	C3, C4, C12, C13, and C17	C12, C13, and C17	C16, C17, C18, and C19	C16, C18, and C19
Cybersecurity awareness	C3, C5, C11, and C12	C3, C5, C11, C12, and C17	C11, C12, and C17	C16, C17, and C18	C16 and C18
Data privacy and protection awareness	C4, C5, C11, and C13	C4, C5, C11, C13, and C17	C11, C13, and C17	C16, C17, and C19	C16 and C19
Cybersecurity training	C1, C2, C7, C8, and C11	C7, C8, C11, C12, and C17	C11, C12, C14, and C17	C14, C16, C17, and C18	C16 and C18
Data privacy and protection training	C1, C2, C9, and C10	C9, C10, C11, C13, and C17	C11, C13, C15, and C17	C15, C16, C17, and C19	C16 and C19
Communication channels	C3, C4, and C5	C3, C4, C5, and C17	C14, C15, and C17	C14, C15, and C17	N/A^a^
Secure connection and use of devices	C3, C4, C5, C6, C9, and C10	C3, C4, C5, C6, C9, C10, and C17	C10, C14, C15, and C17	C14, C15, C16, C17, C18, and C19	C16, C18, and C19

^a^N/A: not applicable.

### Example Application of the Proposed Risk-Based Survey Analysis Approach

In this section, we provide an example application of the proposed risk-based approach to demonstrate, in a simple way, how it works in practice. In this example, we consider the administrative employee group at one of the CUREX partner health care organizations.

First, the risk category is given with the corresponding number of questions in Table 8. The types of these specific questions are agreement (ie, questions 3 and 4) and awareness—*yes, no, or I don’t know* (ie, question 2). “Re” represents the number of responses for each case, and “Marks” represents the corresponding mark.

Thus, the first step is the collection of responses and rating them using the scoring system from 1 to 5 for this risk category. Next, we calculate the risk marking by multiplying *Re* by *Marks*. Then, we sum them all up and multiply the result by the corresponding risk factor.

Following, we give the risk calculation of the aforementioned example.


Risk marking = (9 × 1) + (11 × 2) + (2 × 3) + (6 × 4) + (2 × 5) + (15 × 5) × RF ≈ 16 є (15-19), where RF = 5 / (3 × 15).

The risk marking, which is rounded to a whole number, is 16. By using the risk evaluation matrix, this risk marking shows that the risk is *Medium-high* and the corresponding risk strategy is *Reduction* (Table 2). In this case, the recommended controls to address and manage the risk related to CH (Table 7) include C3, C4, C12, C13, and C17, where controls C3 and C4, related to cybersecurity and data privacy awareness programs, respectively, can be implemented quarterly or monthly with intermediate-level content.

**Table 8:** Example application of the proposed risk-based survey analysis approach.

Risk category	Survey question	Total questions	Agreement	Re^a^	Marks	“Yes, ” “no, ” or “don’t know”	Re	Marks
Cyber hygiene	2, 3, and 4	3	Strongly agree	9	1	“Yes”	0	1
Cyber hygiene	2, 3, and 4	3	Agree	11	2	N/A^b^	0	2
Cyber hygiene	2, 3, and 4	3	Cannot say	2	3	“don’t know”	0	3
Cyber hygiene	2, 3, and 4	3	Disagree	6	4	N/A	0	4
Cyber hygiene	2, 3, and 4	3	Strongly disagree	2	5	“No”	15	5

^a^Re: number of responses.

^b^N/A: not applicable.

### Application of the Exploratory CH Methodology

#### Overview

This section presents the results of applying the exploratory methodology to the CUREX health care organizations (hospitals and research institutes), including the survey demographics, the risk-based analysis of the survey responses, and our observations. For anonymizing the survey results and findings, the names of the 3 health care organizations were randomized and replaced with health care organization 1 (HO1), health care organization 2 (HO2), and health care organization 3 (HO3). The analysis of the results was performed with regard to 3 different aspects. Specifically, in the following sections, we first present the survey demographics and then report a sample of the results with general remarks and discussion regarding the following dimensions: (1) dimension 1—health care organization (HO2), (2) dimension 2—employee group (medical and clinical), and (3) dimension 3—risk category (cybersecurity awareness).

#### Survey Demographics

The demographics of the survey respondents from the 3 CUREX health care organizations are listed in Table 9, including the employee groups, population size, total number of responses, and CI for HO1, HO2, and HO3.

**Table 9:** Survey demographics for the health care organizations in the Secure and Private Health Data Exchange (CUREX) project.

	HO1^a^			HO2^b^			HO3^c^		
	Population (n=1815), n (%)	Responses (n=71), n (%)	95% CI	Population (n=2771), n (%)	Responses (n=199), n (%)	95% CI	Population (n=632), n (%)	Responses (n=86), n (%)	95% CI
Administrative	278 (15.3)	15 (21.1)	−9.66 to 39.66	24 (0.9)	16 (8)	1.55 to 30.45	78 (12.3)	16 (18.6)	−5.98 to 37.98
Medical and clinical	1437 (79.2)	29 (40.8)	10.98 to 47.02	2730 (98.5)	178 (89.4)	170.9 to185.1	554 (87.7)	70 (81.4)	59.04 to 80.96
Executive and security	88 (4.8)	16 (22.5)	−6.26 to 38.29	12 (0.4)	3 (1.5)	−48.18 to 54.18	—^d^	—	—
IT and technical	12 (0.7)	11 (15.5)	2.09 to 19.91	5 (0.2)	2 (1)	−58.01 to 62.01	—	—	—

^a^HO1: health care organization 1.

^b^HO2: health care organization 2.

^c^HO3: health care organization 3.

^d^Not available.

As observed in Table 9, in some cases, the total number of responses was much smaller compared with the population size. As a result, the CI for some of the employee groups was not small enough.

Note that, for the last organization (HO3), there were no responses from the executive and security and IT and technical groups; therefore, they were not included in this table.

## Discussion

### Analysis of Results for Dimension 1—Health Care Organization

**Figure 3:**
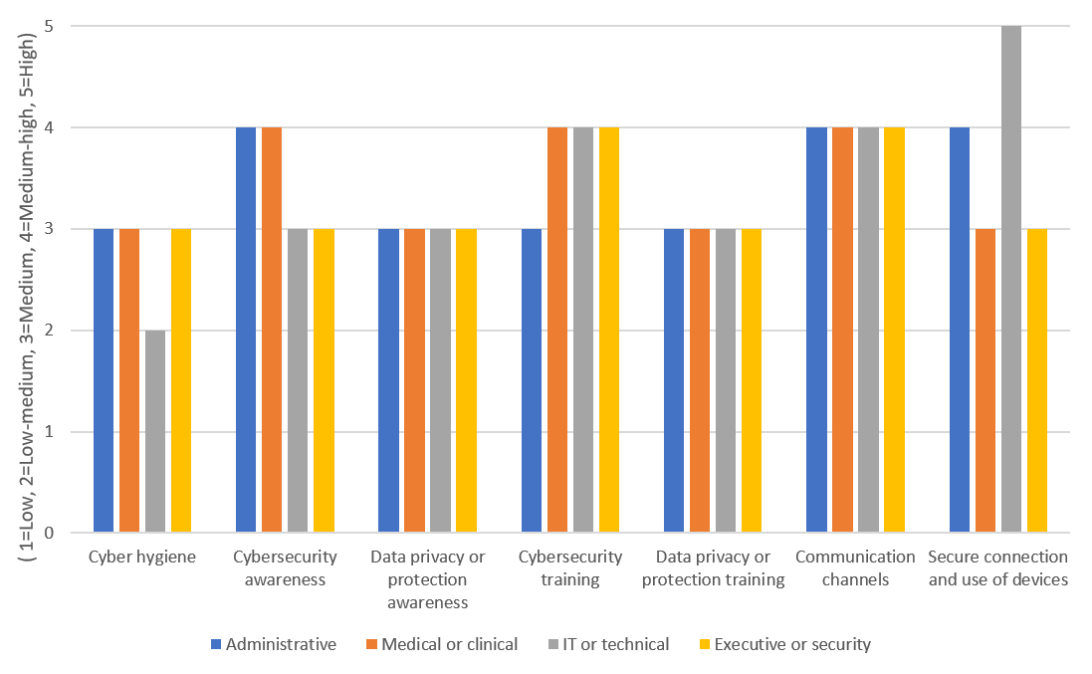
Findings for all employee groups at health care organization 2.

Figure 3 illustrates the results of the application of the risk-based approach in our CH methodology for the risk categories pertaining to all employee groups at HO2.

In Figure 3, the x-axis represents the risk categories, whereas the y-axis represents the risk evaluations as low (1), low-medium (2), medium (3), medium-high (4), and high (5). According to the risk evaluation matrix, these risk evaluations point to specific risk strategies and associated controls to manage the underlying risks.

The results for all employees in Figure 3 indicate that the risks were mostly medium and medium-high, with risk strategies being *Monitoring* and *Reduction*, respectively. Findings for the IT and technical group imply that employees had high awareness of CH as the risk for the corresponding category was medium-low. In contrast, this group demonstrated a high risk for the risk category “Secure Connection and use of devices,” which means that controls should be applied to properly manage this risk compared with the other 3 employee groups. Specifically, based on Table 7, to address this risk, controls C3, C4, C5, C6, C9, and C10 need to be customized with respect to their frequency and content (if applicable) and then targeted to this specific group, as shown in Table 10.

**Table 10:** Subset of human-centric controls for the IT and technical group at health care organization 2.

Number	Control title	Implementation level	
		Frequency	Content level
C3	Implement a cybersecurity awareness program	Monthly or weekly	Beginner
C4	Implement a data privacy awareness program	Monthly or weekly	Beginner
C5	Update awareness content frequently	Monthly or weekly	N/A^a^
C6	Train workforce on secure authentication	Monthly or weekly	Beginner
C9	Train workforce on sensitive data handling	Monthly or weekly	Beginner
C10	Train workforce on causes of unintentional data exposure	Monthly or weekly	Beginner

^a^N/A: not applicable.

### Analysis of Results for Dimension 2—Employee Group

**Figure 4:**
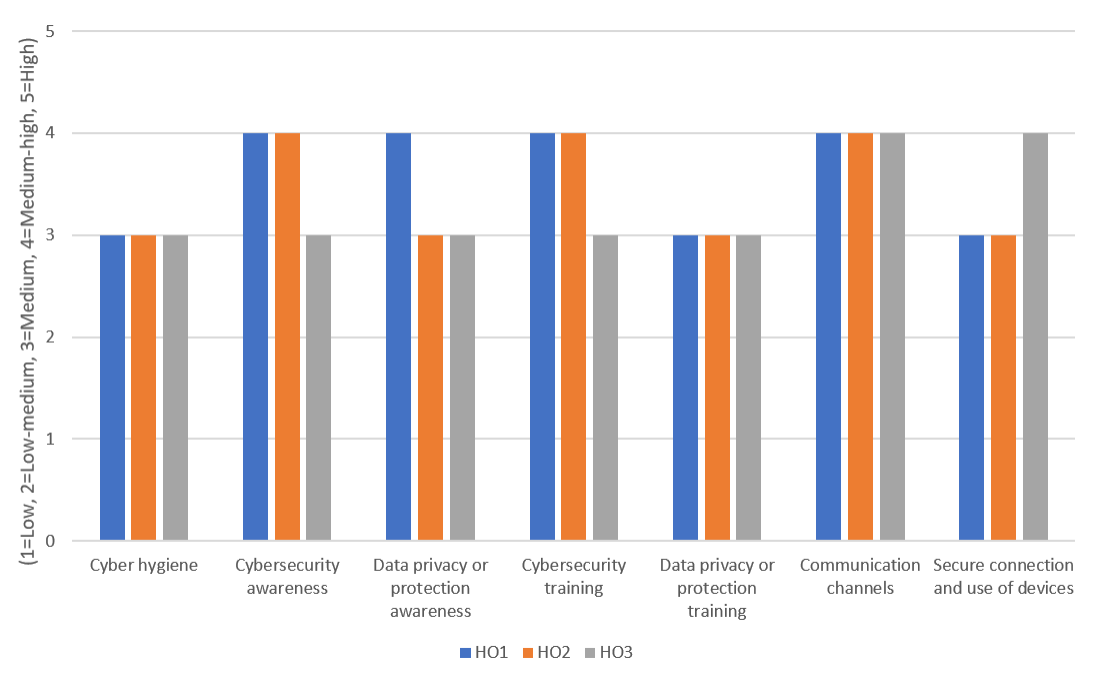
Findings for the medical and clinical employee group at the Secure and Private Health Data Exchange (CUREX) health care organizations. HO1: health care organization 1; HO2: health care organization 2; HO3: health care organization 3.

In Figure 4, survey findings are presented for the medical and clinical employee group at HO1, HO2, and HO3. Similar to the previous graph, the x-axis represents the risk category of the corresponding employee group, and the y-axis represents the risk evaluation.

Findings for this employee group show that the *Cyber hygiene* and *Data Privacy and Protection Training* risk categories had the lowest risk (medium), which needs to be monitored with mild controls. In contrast, the *Communication Channels* risk category had the highest risk as this employee group across all 3 CUREX health care organizations reached a medium-high risk. For this risk, the corresponding controls are C3, C4, C5, and C17, which need to be applied on a quarterly or monthly basis with intermediate-level content for the employees to be able to follow the communication channels and absorb the awareness messages. Moreover, the health care organizations could consider using additional channels for conveying cybersecurity and data privacy messages (eg, channels that are preferable to employees and are not currently in use). As a last observation, employees at HO3 showed lower risks compared with those at HO1 and HO2 as most of their risks were at the medium level.

### Analysis of Results for Dimension 3—Risk Category

**Figure 5:**
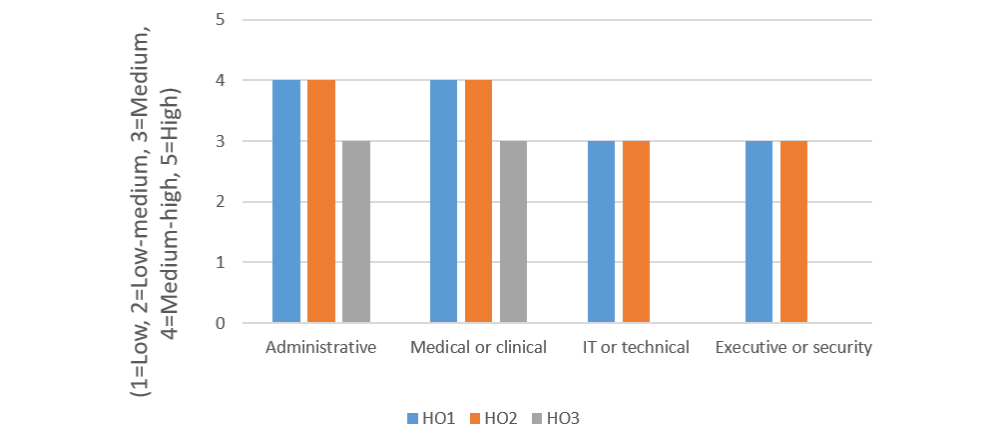
Findings for the cybersecurity awareness risk category at the Secure and Private Health Data Exchange (CUREX) health care organizations. HO1: health care organization 1; HO2: health care organization 2; HO3: health care organization 3.

The bar chart in Figure 5 presents the findings for the 3 CUREX health care organizations with respect to the “Cybersecurity Awareness” risk category pertaining to all employee groups.

The “Cybersecurity Awareness” risk category seemed to have a relatively high evaluation for all employees across the CUREX health care organizations. Specifically, the risk was medium-high at HO1 and HO2 for the administrative, and medical and clinical personnel. The rest of the risk evaluations were at a medium level. The medium-high risks pointed to the *Reduction* risk strategy, where the controls include C3, C5, and C11, which need to be applied every month or quarter with awareness and training content at an intermediate level, as well as controls C12 and C17 for motivating desirable cybersecurity behaviors (Table 7).

### Limitations

The research presented in this paper was conducted across 3 health care organizations. Therefore, the development of the CH controls was based on the limited feedback gathered from the study participants. In addition, as the study period coincided with the COVID-19 pandemic, the responses obtained were limited in number, as outlined in Table 9 (ie, 356 respondents). HO1, HO2, and HO3 had 3.91% (71/1815), 7.18% (199/2771), and 13.6% (86/632) of responses completed from the overall population, respectively. Among the 4 employee groups, the medical and clinical group was represented by 41% (29/71), 89.4% (178/199), and 81% (70/86), respectively, across the health care organizations. It is important to note that 2 employee categories in HO3 were not available to participate in this study. However, the inclusion of 3 different health care organizations brings together different perspectives on cybersecurity and data privacy as experienced by different personnel in the health care sector and paves the way for a CH methodology to recommend targeted human-centric controls. Future work could lead to the analysis of increased responses from geographically diverse groups of health care organizations to further validate the proposed CH controls. We also plan to monitor the application of the recommended controls (ie, step 5 in Figure 1) at a specific health care organization and run the CH survey again after some time to confirm that the situation in terms of cybersecurity and data privacy awareness has improved.

### Comparison With Prior Work

Regarding the literature review, the findings of the study by Cain et al [27] suggest that knowledge about CH is not the same among different age groups, and older users tend to have more secure habits. In the proposed approach, instead of considering the age of the employees, we consider the role of different employees, leading to the identification of 4 employee groups in health care organizations that have different backgrounds and needs regarding CH, not so much because of their age but because of the nature of their work and daily tasks. Considering the findings of the studies by Ashenden and Lawrence [28] and Vishwanath et al [33] related to the use of rewards for encouraging and motivating employees to adopt desirable behaviors, targeted motivation and reward controls were included in the pool of candidate human-centric controls that are recommended to address specific risks. Finally, as phishing emails (and social engineering in general) have been recognized as a serious threat in several studies [27,28,33,34], the proposed methodology focuses on this aspect. The survey questionnaire included questions for different employee groups related to this popular form of social engineering attack as well as specific controls, including training the workforce to identify social engineering attacks and conducting mock social engineering exercises.

### Conclusions

In this paper, a novel concept for improving the CH perception and behavior of 4 key employee groups within health care organizations was proposed. The value of the proposed exploratory survey-based CH methodology was demonstrated through its application to 3 health care organizations that participated in the study in the context of the Horizon 2020 CUREX project. In particular, the proposed CH methodology relies on a survey questionnaire to achieve a deep understanding of the needs and gaps of different health care employee groups. It then uses a risk-based approach to quantify the risk associated with various human-related cybersecurity and data privacy threats, identifies the proper strategies for addressing various risks, and recommends subsets of human-centric controls for managing each risk.

## Appendix

Multimedia Appendix 1 Survey questionnaire.

## Abbreviations

CH: cyber hygiene
CIS: Center for Internet Security
CUREX: Secure and Private Health Data Exchange
HO: health care organization
HO1: health care organization 1
HO2: health care organization 2
HO3: health care organization 3
