# Supplementary material for: Cyber Hygiene Methodology for Raising Cybersecurity and Data Privacy Awareness in Health Care Organizations: Concept Study
Source: J Med Internet Res. 2023 Jul 27;25:e41294. doi: 10.2196/41294 (PMC10415935; doi:10.2196/41294)
Supplement: Multimedia Appendix 1 [file jmir_v25i1e41294_app1.docx]

**Appendix: Survey questionnaire**

The questions of the survey are included in this appendix.

**Legend**

- **Indicates a single-answer question**
- **Indicates a multiple-answer question**

1. What is your role in the organisation?

- Administrative (e.g., administration manager, secretary, reception, call centre, human resources, etc.)
- Medical/Clinical/Research (e.g., department/unit manager, doctor, nurse, researcher, etc.)
- IT/Technical (e.g., IT manager, IT staff, software developer, etc.)
- Executive/Security (e.g., Director, Sub Director, Hospital Manager, Chief Information Officer, Chief Data/Information Security Officer, Data Protection Officer, etc.)

1. Are you familiar with the term Cyber Hygiene?

- Yes
- No

1. To what extend do you agree with the following description of Cyber Hygiene?

(1 = I strongly disagree | 5 = I strongly agree)

*Cyber Hygiene refers to activities that users and computer system administrators can undertake to improve their cybersecurity while online.*

1 2 3 4 5

1. To what extend do you agree with the following description of Cyber Hygiene?

(1 = I strongly disagree | 5 = I strongly agree)

*Cyber Hygiene, in analogy to personal hygiene, refers to simple routine measures that any employee can take to minimise the risks from cyber threats.*

1 2 3 4 5

1. How familiar are you with the General Data Protection Regulation (GDPR)?

(1 = I have never heard of GDPR | 5 = I have in-depth knowledge of GDPR)

1 2 3 4 5

1. Which of the following statements best describes what the GDPR is?

- A new legal framework relating to the collection, storage and usage of personal data, which applies to any organisation based in the EU doing business with EU citizens
- A new legal framework aimed at companies operating online in the EU, stipulating how and when companies are able to collect personal data
- An update on the EU Data Protection Act 1998, which means personal data can only legally be collected and stored by companies that are certified in accordance with the GDPR regulations

1. Who is responsible for monitoring data protection in your business?

- Senior management
- Legal department
- IT managers
- All users of data within the workplace
- Other. Please specify:__________
- I do not know

1. Which of the following cybersecurity and data privacy threats are you aware of? (Select all that apply)

- Social engineering
- Ransomware
- Loss or theft of hardware
- Insider, accidental, or intentional data loss
- Attacks against smart medical devices
- Other. Please specify:__________
- None of the above

1. Have you received any training by your organisation on cybersecurity?
   - Yes. Please note frequency (e.g., Weekly, Monthly, Quarterly, etc.): _______
   - No
2. Have you received any training by your organisation on data privacy?
   - Yes. Please note frequency (e.g., Weekly, Monthly, Quarterly, etc.): _______
   - No
3. Have you heard of any cybersecurity incident outside your organisation (e.g., from the news, etc.)?

- Yes
- No

1. Have you heard of any data privacy incident outside your organisation (e.g., from the news, etc.)?

- Yes
- No

1. Have you ever personally experienced any cybersecurity incident inside your organisation?

- Yes
- No

1. Have you ever personally experienced any data privacy incident inside your organisation?

- Yes
- No

1. Is there a procedure in place in your organisation, in case you face a cybersecurity threat?

- Yes
- No
- I do not know
- Only for specific threats. Please specify: ________________________

1. Is there a procedure in place by your organisation, in case that you face a data privacy threat?

- Yes
- No
- I do not know
- Only for specific threats. Please specify: ________________________

1. How often do you consider cybersecurity during your daily work?

(1 = Never | 5 = In every daily activity)

1 2 3 4 5

1. How often do you consider data privacy during your daily work?

(1 = Never | 5 = In every daily activity)

1 2 3 4 5

1. How often do you manage personal data (i.e. of patients, clients)?

- Never
- Rarely
- Daily
- Weekly
- Monthly

1. How would you rate your knowledge about matters of cybersecurity?

(1 = I have no knowledge | 5 = I am an expert)

1 2 3 4 5

1. How would you rate your knowledge about matters of data privacy?

(1 = I have no knowledge | 5 = I am an expert)

1 2 3 4 5

1. Which communication channels are currently used in your organisation to raise awareness on cybersecurity and data privacy?

(Select all that apply)

- - Emails
  - Corporate Intranet
  - Articles
  - Videos
  - Online training
  - In-person training
  - Information sessions during staff meetings
  - Other. Please specify: ____________________________________
  - I do not know

1. Which communication channels would you prefer to learn about cybersecurity and data privacy in your organisation?

(Select all that apply)

- - Emails
  - Corporate Intranet
  - Articles
  - Videos
  - Online training
  - In-person training
  - Information sessions during staff meetings
  - Other. Please specify: ____________________________________
  - I do not know

1. How often do you interact with your organisation’s IT department or local IT manager?

(1 = Never | 5 = Daily)

1 2 3 4 5

1. Do you personally use remote connection (e.g., Virtual Private Network – VPN) to access your organisation’s corporate network?

- Yes
- No
- I do not know

If yes:

- How often do you do this over public access networks (e.g., public Wi-Fi hotspots)? (1 = Never | 5 = Daily)

1 2 3 4 5

- I am cautious about using public wireless networks.

(1 = I strongly agree | 5 = I strongly disagree)

1 2 3 4 5

1. Does your organisation provide public Wi-Fi access to patients and visitors?

- Yes
- No
- I do not know

1. Is there a Bring Your Own Device (BYOD) policy in your organisation?

- Yes
- No
- I do not know

If yes:

- Did you receive any special training or instructions on this?
- Yes
- No
- I do not know
- I am conscious about protecting my mobile devices and their contents. (1 = I strongly disagree | 5 = I strongly agree)

1 2 3 4 5

1. Are employees allowed to plug in personal USB storage devices on workplace PCs and machines?

- Yes
- No
- I do not know
